# MEST-C pathological score and long-term outcomes of child and adult patients with Henoch-Schönlein purpura nephritis

**DOI:** 10.1186/s12882-020-1691-5

**Published:** 2020-01-30

**Authors:** Donghwan Yun, Dong Ki Kim, Kook-Hwan Oh, Kwon Wook Joo, Kyung Chul Moon, Yon Su Kim, Kyoungbun Lee, Seung Seok Han

**Affiliations:** 1 Department of Internal Medicine, Seoul National University College of Medicine, 103 Daehakro, Jongno-gu, Seoul, 03080 South Korea; 2 Department of Biomedical Sciences, Seoul National University College of Medicine, Seoul, South Korea; 3 Department of Pathology, Seoul National University College of Medicine, 103 Daehakro, Jongno-gu, Seoul, 03080 South Korea

**Keywords:** End-stage renal disease, Henoch-Schönlein purpura nephritis, MEST-C, Oxford classification

## Abstract

**Background:**

Henoch-Schönlein purpura nephritis (HSPN), a small-vessel vasculitis, shares renal pathological features with immunoglobulin A nephropathy. Oxford classification of immunoglobulin A nephropathy pathology has been updated to the MEST-C score, but its application in HSPN remains unresolved.

**Methods:**

Two hundred and thirteen patients with biopsy-proven HSPN were retrieved from the Seoul National University Hospital between 2000 and 2017. Renal outcome risks (i.e., end-stage renal disease or doubling of serum creatinine) were evaluated according to MEST-C scores after stratification by age: 113 children aged < 18 years (9.2 ± 3.6 years) and 100 adults aged ≥18 years (38.6 ± 18.3 years). We pooled our data with four previous cohort studies in which MEST or MEST-C scores were described in detail.

**Results:**

Twenty-one child (19%) and 16 adult (16%) patients reached the renal outcome during the median follow-up periods of 12 years and 13 years, respectively (maximum 19 years). In children, M1 and T1/T2 scores revealed worse renal outcomes than did M0 and T0 scores, respectively, whereas the T score was the only factor related to worse outcomes in adult patients after adjusting for multiple clinical and laboratory variables. The pooled data showed that M1, S1, and T1/T2 in children and E1 and T1/T2 in adults were correlated with poorer renal outcomes than those of their counterpart scores.

**Conclusions:**

The Oxford classification MEST-C scores can predict long-term renal outcomes in patients with HSPN.

## Background

Henoch-Schönlein purpura (HSP) is a small vessel vasculitis that features immunoglobulin A (IgA) deposition, mainly in the joints, skin, and kidneys. The incidences of HSP are approximately 20 and 10 per 100,000 person-years in children and adults, respectively [1], wherein the risk is higher in Asians than in other ethnic groups [2]. When the disease affects the kidneys, it is called HSP nephritis (HSPN). HSPN is rare but can potentially cause chronic kidney disease and end-stage renal disease (ESRD). The prevalence of renal progression to chronic kidney disease or ESRD in patients with HSPN ranges from approximately 5–20% in children and 35–69% in adults [3–6], depending on the patient’s ethnic background [2]. Histopathological findings from renal biopsies are frequently accepted as the most predictable factor of renal outcomes [7, 8].

However, the histopathological classification methods for HSPN are inconsistent and depend on clinicians or societies, although two main classifications exist for HSPN: the Meadow classification, which primarily focuses on the degree of mesangial hypercellularity [9] and the International Study Group of Kidney Disease in Children (ISKDC), which uses crescents as an important factor [10]. Which classification is best for HSPN patients remains uncertain because of limited evidence [11], and the above classifications do not consider other pathological features such as endocapillary hypercellularity, and tubular atrophy / interstitial fibrosis.

HSPN shares many pathological characteristics with IgA nephropathy, and these diseases are indistinguishable from each other in terms of renal pathology [12]. The Oxford classification was developed to score the pathological findings of IgA nephropathy. The previous scoring system included mesangial hypercellularity (M), endocapillary hypercellularity (E), segmental sclerosis (S), and tubular atrophy and interstitial fibrosis (T) [13]. The classification was revised in 2016, wherein the crescent score (C) was added to the scoring system. The Oxford classification with these pathological parameters has shown good predictive power in previous studies [14, 15].

Because HSPN and IgA nephropathy share their renal pathology, a few studies have used the Oxford classification score to predict renal outcomes in HSPN [16–19]. However, these studies yielded inconsistent results for MEST-C score predictability; thus, further study is warranted. The present study addressed the value of the recently updated MEST-C score in predicting long-term renal outcomes in patients with biopsy-proven HSPN. This issue was analyzed after stratification by child vs adult patients.

## Methods

### Patient and data collection

The institutional review board of Seoul National University Hospital approved the study design (no. H-1808-011-963), which was conducted in accordance with the principles of the Declaration of Helsinki. Two hundred and twenty-four patients with clinical features including palpable purpuric eruption and abdominal or joint pain were diagnosed with HSPN via kidney biopsy between 2000 and 2017 years at the Seoul National University Hospital. We excluded 11 patients whose renal pathology slides were unavailable. Consequently, 213 patients were analyzed in the present study. These patients comprised 113 children (aged < 18 years) and 100 adults (aged ≥18 years). Under the review board’s approval, informed consent was waived.

Clinical data on age, sex, body mass index, diabetes mellitus, hypertension, and treatments were collected. Blood parameters, including white blood cell counts, hemoglobin, blood urea nitrogen, creatinine, and cholesterol, were obtained. Proteinuria and hematuria on dipstick tests were scored from negative to ≥3+. The estimated glomerular filtration rate (eGFR) was calculated using the bedside Schwartz equation [20] for children and the Chronic Kidney Disease Epidemiology Collaboration equation [21] for adults.

All kidney biopsy slides were reviewed by nephropathologists and scored according to the revised Oxford classification criteria: mesangial hypercellularity (M0 < 50%; M1 ≥ 50% of the glomeruli), endocapillary hypercellularity (E0: absent; E1: present), segmental glomerulosclerosis (S0: absent; S1: present), tubular atrophy and interstitial fibrosis (T0: 0–24%; T1: 25–49%; and T2: ≥50% of the cortical area), and cellular or fibrocellular crescents (C0: absent; C1: 1–24%; and C2: ≥25% of the glomeruli) [14].

### Study outcome

The primary outcome was either doubling of the baseline serum creatinine or development of ESRD during the follow-up period. ESRD events were obtained from the Kidney Renal Registry database of South Korea. Information on all-cause mortality was identified from the National Database of Statistics, Korea.

### Statistical analysis

Statistical analyses were performed using SPSS (version 23.0; IBM Corp., Armonk, NY, USA) and R software (version 3.5.1; The Comprehensive R Archive Network: http://cran.r-project.org). Categorical and continuous variables are expressed as proportions and the means ± standard deviation for normally distributed variables and as the median with interquartile range for not normally distributed variables. The normality of the distribution was analyzed via the Kolmogorov-Smirnov test. The chi-square test was used to compare categorical variables (Fisher’s exact test if not applicable). The Student’s t-test or the Mann-Whitney *U* test was used to compare continuous variables with or without normal distributions, respectively. Kaplan-Meier survival curves were constructed and compared using the log-rank test. A Cox proportional hazards regression model was applied to calculate hazard ratios (HRs) of the outcome risks. Pooled estimates of the relative risks and 95% confidence intervals were evaluated using the Mantel-Haenszel fixed-effects model if there was no evidence of heterogeneity or the DerSimonian and Laird random effects model if there was heterogeneity between studies. Heterogeneity was assessed using the Cochran Q statistic and I^2^. All *P* values were two-sided, and values < 0.05 were considered significant.

## Results

### Baseline characteristics

In children, the mean age was 9.2 ± 3.6 years, and 46.0% were girls. The median values of the serum creatinine and eGFR at the time of biopsy were 0.6 mg/dL (0.5–0.8 mg/dL) and 111.4 mL/min/1.73 m^2^ (82.4–142.7 mL/min/1.73 m^2^), respectively. Proteinuria and hematuria were present in 83.2 and 91.2% of patients, respectively. In adults, the mean age was 38.6 ± 18.3 years, and 40.0% were women. At the time of biopsy, the median values of the serum creatinine and eGFR were 1.0 mg/dL (0.8–1.2 mg/dL) and 94.6 mL/min/1.73 m^2^ (59.5–108.8 mL/min/1.73 m^2^), respectively. Proteinuria was present in 83.0%, and hematuria was present in 89.0%. The median follow-up periods were 12 and 13 years for children and adults, respectively. Table 1 shows other baseline characteristics of the patients.

**Table 1:** Baseline characteristics of the study patients

	Child (*n* = 113)	Adult (*n* = 100)
Age (years)	9.2 ± 3.6	38.6 ± 18.3
Female (%)	46.0	40.0
Body mass index (kg/m^2^)	18.6 ± 3.7	23.3 ± 3.3
Diabetes mellitus (%)	0	8.1
Hypertension (%)	2.3	8.1
Laboratory findings		
WBC (×10^3^/μL)	10.2 (7.4–12.7)	7.5 (5.9–9.7)
Hemoglobin (g/dL)	12.6 (11.3–13.4)	12.9 (10.7–14.0)
BUN (mg/dL)	12 (10–15)	14 (11–20)
Creatinine (mg/dL)	0.60 (0.45–0.80)	1.00 (0.80–1.24)
eGFR (mL/min/1.73 m^2^)	111.4 (82.4–142.7)	94.6 (59.5–108.8)
Cholesterol (mg/dL)	219 (182–262)	177 (150–202)
Proteinuria (%)		
– or ±	16.8	17.0
1+	5.3	13.0
2+	23.0	35.0
≥ 3+	54.9	35.0
Hematuria (%)		
– or ±	8.8	11.0
1+	8.0	13.0
2+	23.9	19.0
≥ 3+	59.3	57.0
Treatment (%)^a^		
ACEi/ARB	96.5	59.7
Steroid	82.6	54.8
Cytotoxic agents	43.0	6.5

Data are presented as proportion or mean ± standard deviation or median (interquartile range)

*WBC* white blood cells, *BUN* blood urea nitrogen, *eGFR* estimated glomerular filtration, *ACEi* angiotensin converting enzyme inhibitor, *ARB* aldosterone II receptor blocker

^a^Not available in 27 child and 38 adult patients

### Pathological findings according to classification criteria

Using the Oxford classification, M1, E1, S1, T1/T2, and C1/C2 occurred in 54.9, 61.9, 63.7, 5.3% (T1, 4.4%; T2, 0.9%), and 44.2% (C1, 38.9%; C2, 5.3%) of the children, respectively. M1, E1, S1, T1/T2, and C1/C2 occurred in 31.0, 48.0, 59.0, 11.0% (T1, 8.0%; T2, 3.0%) and 38% (C1, 30.0%; C2, 8.0%) of the adults, respectively (Table 2). The rates of M1 (54.9% vs. 31.0%; *P* < 0.001) and E1 (61.9% vs. 48.0%; *P* = 0.041) differed between the children and adults, whereas the other scores did not.

**Table 2:** Renal pathology findings

Oxford classification (%)	Child patients				Adult patients			
	Total	Non-progression	Progression	*P*	Total	Non-progression	Progression	*P*
M1	54.9	48.9	81.0	0.008	31.0	28.6	43.8	0.249
E1	61.9	58.7	76.2	0.136	48.0	46.4	56.3	0.471
S1	63.7	62.0	71.4	0.415	59.0	59.5	56.3	0.807
T1/T2	5.3	3.3	14.3	0.042	11.0	6.0	37.5	0.002
C1/C2	44.2	44.6	42.9	0.887	38.0	34.5	56.3	0.101

*M* mesangial hypercellularity, *E* endocapillary hypercellularity, *S* segmental glomerulosclerosis, *T* tubular atrophy and interstitial fibrosis, *C* cellular or fibrocellular crescents

### Renal outcomes according to classification

During the follow-up period, 21 children (18.6%) and 16 adults (16.0%) reached the primary endpoint of serum creatinine doubling or the ESRD event. Among them, 4 children and 7 adults progressed to ESRD. The treatment regimens did not differ between the progression and non-progression groups (Additional file 1: Table S1). Children in the progression group had higher rates of M1 and T1/T2 than did those in the non-progression group. In adults, only T scores were higher in the progression group but not the non-progression group in adults. The all-cause mortality rates were 0 and 16% in children and adults, respectively.

**Fig. 1:**
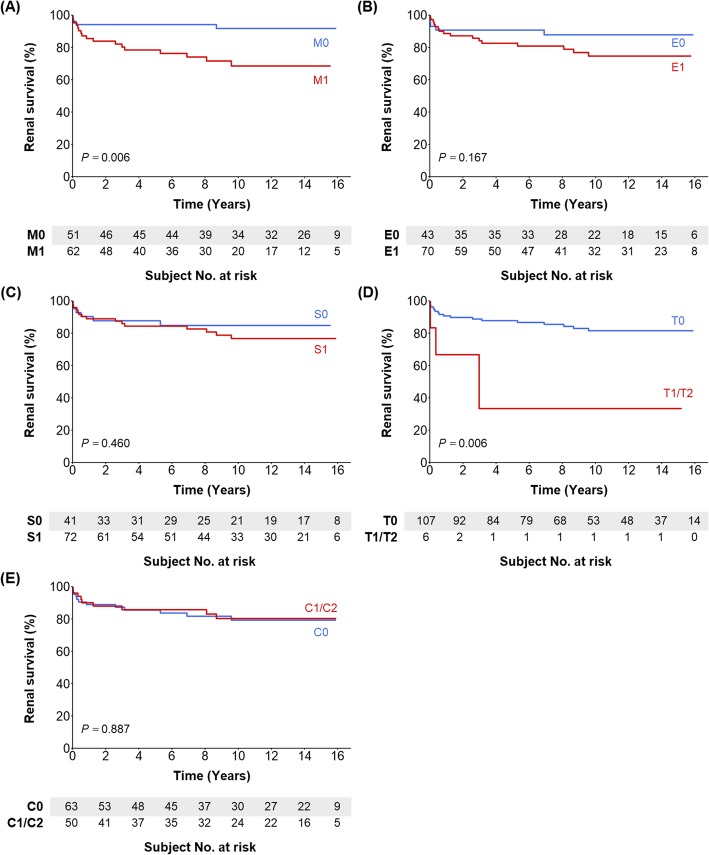
Renal outcome-free survival curves according to MEST-C scores in child patients. **a** M0 vs. M1; **b** E0 vs. E1; **c** S0 vs. S1; **d** T0 vs. T1/T2; **e** C0 vs. C1/C2

**Fig. 2:**
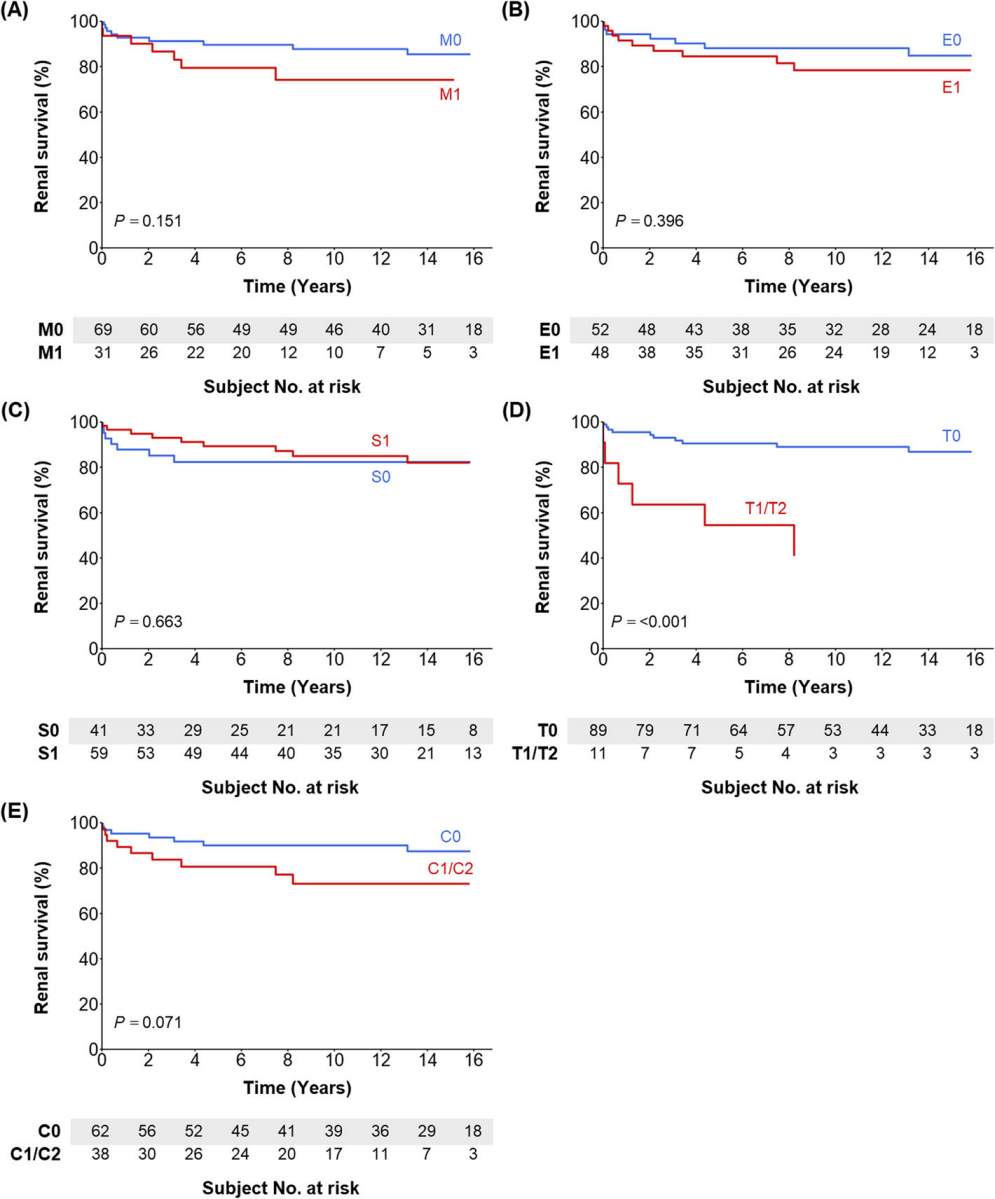
Renal outcome-free survival curves according to MEST-C scores in adult patients. **a** M0 vs. M1; **b** E0 vs. E1; **c** S0 vs. S1; **d** T0 vs. T1/T2; **e** C0 vs. C1/C2

Figure 1 (child patients) and Fig. 2 (adult patients) present the renal outcome-free curves according to MEST-C score. Children with M1 and T1/T2 had poorer renal outcomes than did those with M0 and T0, respectively. The other scores did not separate the survival curves. In the adult group, T1 and T2 were associated with poorer renal outcomes compared with T0. Patients with C1/C2 had poorer outcomes than did those with C0, although the significance was marginal. The other scores did not correlate with renal outcomes. Table 3 show the unadjusted and adjusted Cox models in children and adults. In model 1 (the univariate model), the M1 and T1/T2 scores for children and the T1/T2 scores for adults were associated with worse renal outcomes. Multivariate models 2–4 also showed that M1 and T1/T2 for children and T1/T2 for adults were independently associated with a risk of poor renal outcome.

**Table 3:** Prediction of renal outcomes among MEST-C scores

Parameters	HR (95% CI) from Model 1	*P*	HR (95% CI) from Model 2	*P*	HR (95% CI) from Model 3	*P*	HR (95% CI) from Model 4	*P*
Child patients								
M1 (vs. M0)	4.16 (1.394–12.407)	0.011	3.38 (1.101–10.363)	0.033	3.53 (1.118–11.140)	0.032	4.02 (1.217–13.304)	0.023
E1 (vs. E0)	2.00 (0.734–5.470)	0.175	2.14 (0.720–6.355)	0.171	1.56 (0.486–5.026)	0.453	1.50 (0.464–4.832)	0.499
S1 (vs. S0)	1.43 (0.553–3.675)	0.463	1.44 (0.554–3.762)	0.452	1.36 (0.508–3.642)	0.540	1.35 (0.494–3.672)	0.560
T1/T2 (vs. T0)	4.86 (1.406–16.794)	0.012	4.24 (1.124–15.979)	0.033	9.74 (1.920–49.462)	0.006	12.28 (2.728–55.273)	0.001
C1/C2 (vs. C0)	0.94 (0.396–2.229)	0.887	0.60 (0.232–1.569)	0.300	0.59 (0.231–1.524)	0.279	0.58 (0.216–1.531)	0.268
Adult patients								
M1 (vs. M0)	2.05 (0.756–5.532)	0.159	1.29 (0.420–3.933)	0.659	1.41 (0.411–4.819)	0.586	1.36 (0.410–4.473)	0.618
E1 (vs. E0)	1.53 (0.569–4.114)	0.399	0.86 (0.233–3.170)	0.820	0.56 (0.139–2.239)	0.410	0.35 (0.075–1.598)	0.174
S1 (vs. S0)	0.80 (0.299–2.159)	0.664	1.01 (0.354–2.907)	0.979	1.15 (0.371–3.547)	0.812	1.02 (0.357–2.898)	0.974
T1/T2 (vs. T0)	6.25 (2.265–17.258)	< 0.001	5.32 (1.807–15.660)	0.002	4.29 (1.124–16.333)	0.033	4.02 (1.665–9.684)	0.002
C1/C2 (vs. C0)	2.43 (0.900–6.553)	0.080	1.95 (0.494–7.717)	0.340	1.57 (0.389–6.334)	0.526	0.85 (0.198–3.639)	0.825

*HR* Hazard ratio, *CI* confidence interval

Model 1: Unadjusted

Model 2: Adjusted for other pathological scores

Model 3: Adjusted for model 2 plus age, sex, and estimated glomerular filtration rate

Model 4: Adjusted for model 3 plus proteinuria and hematuria

Because all child patients were alive during the follow-up period, the risk of all-cause mortality was evaluated only in adults. E1 and C1/C2 were significant variables in the unadjusted Cox model, but no variable predicted the risk of mortality after adjusting for multiple clinical variables (Additional file 2: Table S2).

### Pooled analysis with previous studies

**Fig. 3:**
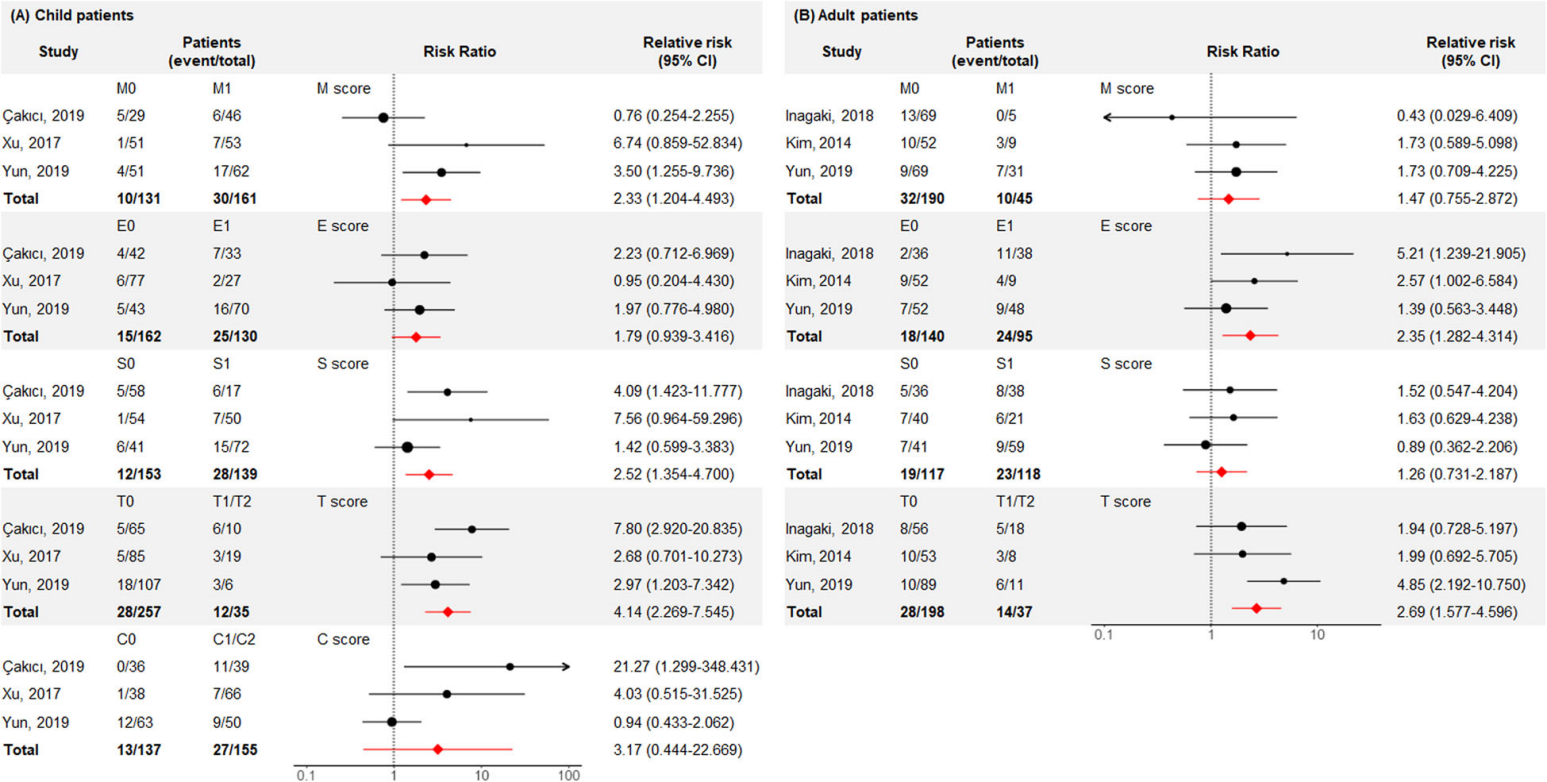
Pooled analysis of renal outcome according to MEST-C scores in children (**a**) and MEST scores in adults (**b**)

Pooled analysis of the predictability of MEST and MEST-C scores for HSPN patients was performed using four previous studies [16–19], although the relationship for C score in adults could not be determined because the C score was defined differently in one study [19]. Two hundred ninety-two children and 235 adults were included in the meta-analysis, and their information is summarized in Table 4. In the children, M1, S1, and T1/T2 scores correlated with poor renal outcomes, while E1 and T1/T2 were associated with worse renal outcomes in adults (Fig. 3).

**Table 4:** Summary of studies regarding application of the Oxford classification to patients with Henoch-Schönlein purpura nephritis

Study	No. of patients	Age	Definition of renal outcome	Significant pathologic parameters	
				Univariate model	Multivariate model
Child patients					
Çakıcı et el. (2019) [17]	75	< 18 years	< 90 mL/min/1.73 m^2^ or 50% decrease of eGFR; or persistent PU/HU	S1, T1/T2	T1/T2^b^
Xu et el. (2018) [18]	104	< 18 years	< 90 mL/min/1.73 m^2^ or 50% decrease of eGFR	S1	None^c^
The present study	113	< 18 years	ESRD or doubling of sCr	M1, T1/T2	M1, T1/T2
Adult patients					
Inagaki et el. (2018) [16]	74	≥18 years	ESRD or 30% decrease of eGFR	E1	E1^d^
Kim et el. (2014)^a^ [19]	61	≥16 years	ESRD; or < 60 mL/min/1.73 m^2^ or ≥ 30% decrease of eGFR	E1, T1/T2, C^a^	E1, T1/T2^e^
The present study	100	≥18 years	ESRD or doubling of sCr	T1/T2	T1/T2

*ESRD* end-stage renal disease, *sCr* serum creatinine, *eGFR* estimated glomerular filtration rate, *PU* proteinuria, *HU* hematuria

^a^Crescent formation was defined when crescents were ≥ 50% of the glomeruli, which differs from the updated Oxford classification (C1: 0–25%; C2: ≥25%)

^b^Adjusted for E, S, T, and estimated glomerular filtration rate

^c^Adjusted for M, E, S, T, and C

^d^Adjusted for E, T, C, age, sex, and proteinuria

^e^Adjusted for E, T, C, hypertension, proteinuria, and estimated glomerular filtration rate

## Discussion

Renal pathology findings may determine the renal outcomes of HSPN [7, 8]. However, pathological classification of HSPN has been inconsistent between studies [11], and many of the previous pathological classifications provided no information on tubular atrophy and interstitial fibrosis, which has frequently been shown to predict renal outcomes [17, 19, 22, 23]. The updated Oxford classification of IgA nephropathy includes information on various pathological features, which are graded as the MEST-C score; however, applying this classification to HSPN cases requires further validation [14]. The present study found that M and T scores in children and T scores in adults were associated with the renal outcomes of HSPN. A pooled analysis supported these findings.

Increasing evidence shows the benefits of using the Oxford classification. A previous study involving 61 adult patients with HSPN revealed that E1 and T1/T2 were independently associated with poor renal outcomes [19]. Another study found that E1 lesions were the only significant variable related to renal outcomes [16]. A study of 104 children showed that S1 lesions were predictors of renal outcome, and T1/T2 were risk factors for non-remission from proteinuria [18]. A recent study involving 75 children with HSPN found that T1/T2 was an independent predictor of renal outcome, whereas the effect of S1 was dependent on the other variables [17]. Collectively, the E, S, and T scores have been shown to predict renal outcomes in HSPN. The present study first identified the validity of the M score in children with HSPN. Pooling these studies showed that M1, S1, and T1/T2 in child patients and E1 and T1/T2 in adult patients were associated with poor renal outcomes. These findings were similar to or different from the results for IgA nephropathy [15, 24], and several factors, such as underlying pathophysiology, clinical conditions, and treatments, would affect the predictability of each score.

The present study found no evidence that C scores can predict renal outcomes. Many previous studies also failed to demonstrate the predictability of C lesions although these studies did not use the recent Oxford classification [25–27]. Two large Chinese studies, wherein the definition of crescentic severity complied with the Oxford classification, found no independent value of crescents relative to renal outcomes [28, 29]. Another study, which defined crescents as ≥50% of the glomeruli, failed to show that crescents had an independent value in predicting renal outcomes of HSPN [19]. Crescent formation, which is an important pathological feature of the ISKDC classification, was recently added to the Oxford classification of IgA nephropathy and is considered to predict a poor prognosis in Chinese patients with IgA nephropathy [30, 31]. However, in HSPN cases, the C score may need to be adjusted to increase its predictive value or clinical application.

Although the present study results are informative, this study had some limitations. The study used a retrospective design and thus could not determine a causal relationship. The random urine protein-to-creatinine ratio, which is the best parameter of proteinuria, was not considered because half the patients had missing values. Other outcomes, such as proteinuria remission, were not obtained, and these outcomes may have been associated with the MEST-C scores. The use of treatment agents, such as anti-hypertensive drugs and steroids, was not considered in multivariate models because of missing values, although these agents have been known to affect the predictability of pathological parameters [15, 32]. The results from Korean patients may have limited applicability to Caucasian patients; thus, a current pooled analysis with results from both ethnics may be more generalizable than results from Korean patients alone. These issues will be addressed in future studies with larger sample sizes, and a new initiative of the International IgA Nephropathy Network is currently evaluating > 1000 renal biopsies from HSPN [33].

## Conclusion

The MEST-C scores of the Oxford classification, particularly M and T, are related to renal outcomes in patients with HSPN. These results suggest that the pathological classification of IgA nephropathy may also be applied to HSPN patients. Nevertheless, the predictability of certain scores, such as C lesions, should be further evaluated or adjusted in up-coming studies.

## Supplementary information

**Additional file 1: Table S1.** Comparison of treatment regimen between progression and non-progression groups.

**Additional file 2: Table S2.** Prediction of all-cause mortality in adults.

## Data Availability

The datasets used or analyzed during the current study can be obtained on reasonable request with the permission from the corresponding author.

## Abbreviations

eGFR: Estimated glomerular filtration rate
ESRD: End-stage renal disease
HSP: Henoch-Schönlein purpura
HSPN: Henoch-Schönlein purpura nephritis
IgA: Immunoglobulin A
ISKDC: International Study Group of Kidney Disease in Children
