# Supplementary material for: MEST-C pathological score and long-term outcomes of child and adult patients with Henoch-Schönlein purpura nephritis
Source: BMC Nephrol. 2020 Jan 30;21:33. doi: 10.1186/s12882-020-1691-5 (PMC6993338; doi:10.1186/s12882-020-1691-5)
Supplement: Supplementary file 1 — Additional file 1: Table S1. Comparison of treatment regimen between progression and non-progression groups. [file 12882_2020_1691_MOESM1_ESM.docx]

Table S1. Comparison of treatment regimen between progression and non-progression groups

|  | Child patients | | | Adult patients | | |
| --- | --- | --- | --- | --- | --- | --- |
| Treatment | Non-progression  (n = 92) | Progression  (n = 21) | *P* | Non-progression  (n = 84) | Progression  (n = 16) | *P* |
| ACEi/ARB (%) | 96.9 | 95.2 | 1.000 | 59.2 | 61.5 | 0.878 |
| Steroid (%) | 83.1 | 81.0 | 1.000 | 51.0 | 69.2 | 0.241 |
| Cytotoxic agents (%) | 41.5 | 47.6 | 0.800 | 4.1 | 15.4 | 0.191 |

ACEi, angiotensin converting enzyme inhibitor; ARB, aldosterone II receptor blocker.
