# Supplementary material for: MEST-C pathological score and long-term outcomes of child and adult patients with Henoch-Schönlein purpura nephritis
Source: BMC Nephrol. 2020 Jan 30;21:33. doi: 10.1186/s12882-020-1691-5 (PMC6993338; doi:10.1186/s12882-020-1691-5)
Supplement: Supplementary file 2 — Additional file 2: Table S2. Prediction of all-cause mortality in adults. [file 12882_2020_1691_MOESM2_ESM.docx]

Table S2. Prediction of all-cause mortality in adults

| Parameters | HR (95% CI) from Model 1 | *P* | HR (95% CI) from Model 2 | *P* | HR (95% CI) from Model 3 | *P* | HR (95% CI) from Model 4 | *P* |
| --- | --- | --- | --- | --- | --- | --- | --- | --- |
| M1 (vs. M0) | 1.72 (0.620–4.789) | 0.297 | 0.87 (0.290–2.612) | 0.804 | 1.70 (0.545–5.311) | 0.361 | 1.54 (0.472–5.029) | 0.474 |
| E1 (vs. E0) | 5.26 (1.496–18.459) | 0.010 | 3.87 (0.895–16.766) | 0.070 | 0.90 (0.172–4.747) | 0.905 | 0.75 (0.139–4.031) | 0.735 |
| S1 (vs. S0) | 0.47 (0.174–1.257) | 0.132 | 0.46 (0.170–1.254) | 0.130 | 0.38 (0.127–1.166) | 0.091 | 0.41 (0.135–1.223) | 0.109 |
| T1/T2 (vs. T0) | 1.71 (0.487–6.005) | 0.402 | 1.22 (0.306–4.836) | 0.781 | 0.55 (0.108–2.778) | 0.467 | 0.58 (0.105–3.162) | 0.526 |
| C1/C2 (vs. C0) | 3.31 (1.197–9.158) | 0.021 | 1.80 (0.532–6.051) | 0.346 | 1.14 (0.313–4.178) | 0.839 | 0.99 (0.252–3.907) | 0.990 |

HR, Hazard ratio; CI, confidence interval

Model 1: Unadjusted.

Model 2: Adjusted for other pathological scores.

Model 3: Adjusted for model 2 plus age, sex, and estimated glomerular filtration rate.

Model 4: Adjusted for model 3 plus proteinuria and hematuria.

eGFR; estimated glomerular filtration ratio.
